# The genus *Phyllomyza* Fallén from China, with descriptions of three new species (Diptera, Milichiidae)

**DOI:** 10.3897/zookeys.760.22595

**Published:** 2018-05-28

**Authors:** Yu-Qiang Xi, Ding Yang, Xin-Ming Yin

**Affiliations:** 1 Department of Entomology, Henan Agricultural University, No. 95 Wenhua Road, Jinshui District, Zhengzhou 450003, Henan Province, China; 2 Xinyang College of Agriculture and Forestry, 24th Street, Yangshan District, Xinyang 464000, Henan Province, China; 3 Department of Entomology, China Agricultural University, No. 2 Yuanmingyuan West Road, Haidian District, Beijing 100193, China

**Keywords:** China, Milichiidae, Diptera, new species, *Phyllomyza*

## Abstract

The following three species of the genus *Phyllomyza* Fallén from China are described as new to science: *P. guangxiensis* **sp. n.**, *P. luteigenis* **sp. n.**, and *P. quadratpalpus* **sp. n.** A key to the known species of *Phyllomyza* from China is presented.

## Introduction

The genus *Phyllomyza* was established by Fallén in 1810. It belongs to the subfamily Phyllomyzinae of the family Milichiidae and most species are recognized by the following characteristics: three lateroclinate orbital setae; occiput not strongly concave when viewed from above; palpus and first flagellomere in male usually greatly enlarged, longer than broad; lunule usually with a pair of setae (Brake 2000). There are 49 described species distributed widely in the world except the Neotropical region (Malloch 1914a; Hennig 1967; Yang 1998; Brake 2000; Iwasa 2003; Xi and Yang 2013, 2015a, b; Xi et al. 2016). 15 species are known from the Palaearctic region (Hennig 1937; Papp 1976, 1984; Papp and Wheeler 1998; Yang 1998) and 29 species from the Oriental region (Brake 2000; Iwasa 2003; Xi and Yang 2013, 2015a, b; Xi et al.2016). There are 20 species occurring in China, of which four species are distributed in Taiwan (Hendel 1914; Malloch 1914b; Yang 1998; Xi and Yang 2013, 2015a, b; Xi et al. 2016). In the present paper, three species of the genus from China are described as new to science. A key to the described species of *Phyllomyza* from China is presented. Larvae of some *Phyllomyza* are generally saprophagous and live in decaying plants, or rear from nests of ants (Donisthorpe 1927). Adults of some species can be collected in open landscapes, such as steppes or meadow, in wadis, at the edges of forests, inside forests, in the forest canopy, in stables or houses, or even in caves, but they don’t seem to be found in coastal habitats or to other places near water (Brake 2000), this habit is different from Canacidae and Tethinidae, each of which have members with similar physical characters to the genus *Phyllomyza*.

## Materials and methods

Genitalia preparations were made by removing and macerating the apical portion of the abdomen in glacial acetic acid, then rinsed in distilled water brfore being stored in glycerine filled microvials. After examination, they were transferred to fresh glycerine and stored in a microvial on the pin below the specimen or moved to an ethanol tube together with the wet specimens. Specimens examined were deposited in the Entomological Museum of China Agricultural University (**CAU**), Beijing; the Entomological Museum of Henan Agricultural University (**HAU**). The general terminology follows McAlpine (1981) and Brake (2000). The following abbreviations are used:

**asc** apical scutellar seta(e),

**bsc** basal scutellar seta(e),

**dc** dorsocentral seta(e),

**h** humeral seta(e),

**ia** intraalar seta(e),

**kepsts** katepisternal seta(e),

**npl** notopleural seta(e),

**pa** postalar seta(e),

**pos** postsutural seta(e),

**prs** presutural seta(e),

**prsc** prescutellar seta(e),

**sa** supraalar seta(e),

**S** sternite,

**T** tergite.

## Taxonomy

### Key to species (males) of *Phyllomyza* from China

**Table:** 

1	Palpus almost bare, without setulae	**2**
–	Palpus with short setulae at tip and on ventral side	**7**
2	Frons with 3 interfrontal setae	***P. fuscusa* Xi, Yin & Yang**
–	Frons with 4 interfrontal setae	**3**
3	Arista 2.5 times as long as first flagellomere; cercus with or lacking ventral appendix	**4**
–	Arista 3 times as long as first flagellomere; cercus lacking ventral appendix	**5**
4	Gena approximately one-fourth eye height (Fig. 5); cercus lacking ventral appendix (Fig. 7)	***P. luteigenis* sp. n.**
–	Gena approximately one-eighth eye height (Fig. 25); cercus with ventral appendix (Fig. 26)	***P. nudipalpis* Malloch**
5	Gena very narrow, approximately one-fourteenth eye height (Fig. 1); first flagellomere irregularly oblong; knob of halter brownish	***P. guangxiensis* sp. n.**
–	Gena at least one-tenth eye height; first flagellomere irregularly quadrate; knob of halter yellowish	**6**
6	Gena approximately one-sixth eye height; first flagellomere 2 times wider than long	***P. leioipalpus* Xi, Yin & Yang**
–	Gena approximately one-tenth eye height; the length of first flagellomere is the same as width	***P. aureolusa* Xi, Yin & Yang**
7	Palpus very long, at least 6 times as long as wide	**8**
–	Palpus relatively short, less than 5 times as long as wide	**9**
8	First flagellomere long, 1.4 times as long as wide; knob of halter yellowish white	***P. basilatusa* Xi, Yin & Yang**
–	First flagellomere wide, 1.1 times as long as wide; knob of halter darkish brown	***P. sinensis* Xi & Yang**
9	Cercus with ventral appendix (Fig. 22); first flagellomere broadly elliptoid	***P. epitacta* Hendel**
–	Cercus lacking ventral appendix; first flagellomere very broadly oblong	**10**
10	Palpus pointed apically; first flagellomere shallowly panduriform (Figs 15, 16)	***P. claviconis* Yang**
–	Palpus inflated, blunted apically; first flagellomere broadly elliptoid or shallowly oblong	**11**
11	First flagellomere shallowly oblong, 1.3 times as long as wide	***P. quadratpalpus* sp. n.**
–	First flagellomere broadly elliptoid, almost the same length and width	**12**
12	M_1_ between r-m and dm-cu as long as dm-cu	**13**
–	M_1_ between r-m and dm-cu longer than dm-cu	**14**
13	Gena narrow, approximately one-eleventh eye height; palpus 5 times as long as wide (Figs 13, 14)	***P. angustigenis* Xi & Yang**
–	Gena relatively broad, approximately one-seventh eye height; palpus 7 times as long as wide (Figs 23, 24)	***P. euthyipalpis* Xi & Yang**
14	M_1_ between r-m and dm-cu at least 1.5 times longer than dm-cu	**15**
–	M_1_ between r-m and dm-cu less than 1.2 times longer than dm-cu	**19**
15	Vibrissa located at level of lower margin of eye	**16**
–	Vibrissa located below level of lower margin of eye	**17**
16	Palpus yellow; hind tibia yellowish	***P. luteipalpis* Malloch**
–	Palpus dark brown; hind tibia yellow to brown	***P. clavellata* Xi & Yang**
17	Halter white; palpus curved	***P. drepanipalpis* Xi & Yang**
–	Halter dark brown or yellowish; palpus straight	**18**
18	Gena approximately one-seventh eye height; knob of halter yellowish	***P. emeishanensis* Xi & Yang**
–	Gena approximately one-fifth eye height; knob of halter with upper half white and lower half dark brown	***P. latustigenis* Xi & Yang**
19	Gena broad, approximately one-sixth eye height (Figs 19, 20)	***P. dicrana* Xi & Yang**
–	Gena narrow, less than one-sixth eye height	**20**
20	Vibrissal angle relatively acute, the tip less than 60°angle (Figs 17, 18)	***P. cuspigera* Xi & Yang**
–	Vibrissal angle blunt, the tip almost 90°angle	**21**
21	Cercus with thin ventral appendix (Fig. 21); hind tibia brownish	***P. dilatata* Malloch**
–	Cercus lacking short ventral appendix; hind tibia dark brown	**22**
22	Arista approximately 4.5 times as long as first flagellomere; knob of halter with upper half brownish and lower half dark brown (Figs 27, 28)	***P. planipalpis* Xi & Yang**
–	Arista approximately 3.5 times as long as first flagellomere; knob of halter with upper half yellow and lower half yellowish (Figs 29, 30)	***P. tibetensis* Xi & Yang**

#### Phyllomyza guangxiensis sp. n.

Taxon classificationAnimaliaDipteraMilichiidae

http://zoobank.org/FCE8BDB1-7760-434F-BCC8-168358778852

Figs 1–4

##### Diagnosis.

Gena approximately one-fourteenth of eye height. Upper blade of bifurcated tip of surstylus swollen and apical margin rounded, lower blade thin and longer than upper one; cercus arched with short sparse setae.

##### Description.

*Male*. Body length 1.6–1.7 mm; wing length 1.6–1.7 mm.

**Figures 1–4.**
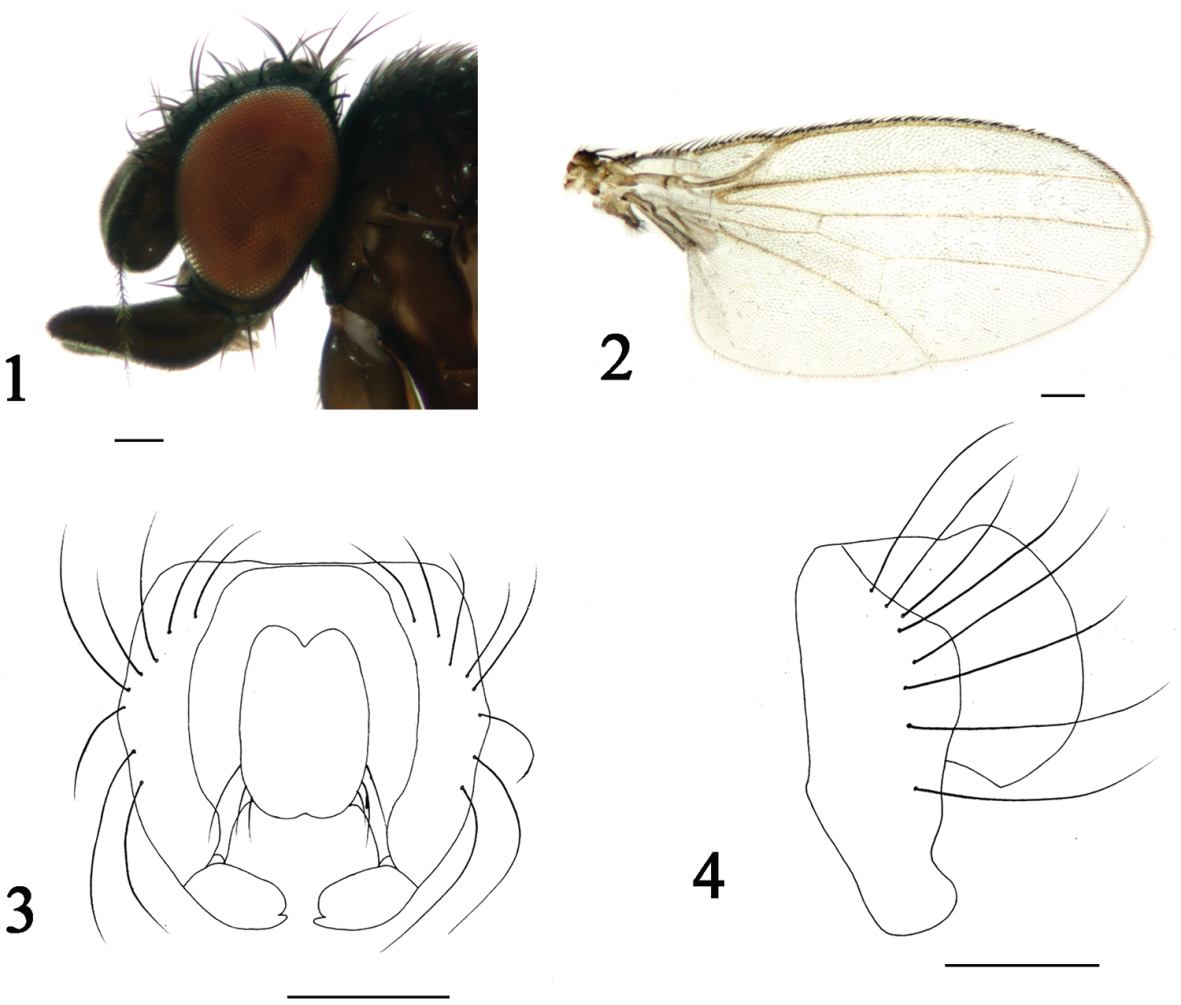
*Phyllomyza guangxiensis* sp. n. (male). **1** Head, lateral view **2** wing **3** epandrium, cerci, and surstyli, posterior view **4** epandrium, cerci, and surstyli, lateral view. Scale bars: 0.1 mm.

Head (Fig. 1) black with greyish microtomentum; orbital plate satiny blackish brown, with microtomentum, ocellar triangle blackish brown without microtomentum; lunule very depressed falciform, darkish brown with black margin. Posterior eye margin ventrally diverging from head margin; eye 1.4 times as high as long, gena approximately one-fourteenth of eye height. Setae and setulae on head black; ocellar triangle with two ocellar setae and three short setae; frons with three orbital and two frontal setae on blackish brown orbital stripe, orbital setae lateroclinate and frontal setae medioclinate, four interfrontal setae; postocellar setae cruciate. Lunule with two setae. Vibrissal angle flat, the tip a little more than a 90°angle; vibrissa strong, located below level of lower margin of eye. Antenna darkish brown pedicel with short black setulae at middle and margin, setulae at margin longer than others, longest one approximately five times longer than others; first flagellomere with pubescence, irregularly oblong; arista three times as long as first flagellomere, black, distinctly pubescent. Proboscis short and folded, darkish brown, with sparse black setulae. Palpus wide, 0.5 mm, with blunt apex in lateral view; darkish brown with short dense black pubescence, margin without sparse setulae.

Thorax brown with grey microtomentum, except scutum shiny brownish with sparse black microtomentum; scutellum brownish with gray microtomentum. Setae and setulae on thorax black; one h, two dc, one prsc, two npl, one prs, one pos, one sa, one pa, one kepsts (setulae at forward position); scutellum 1.5 times wider than long, with pair of asc and bsc, asc three times longer than bsc. Legs slender, coxae and femora darkish brown, tibiae yellow except hind tibia brown with yellow apex, tarsi yellowish. Setae and setulae on legs black, mid tibia with single black preapical dorsal seta. Wing (Fig. 2) hyaline, unspotted; veins brown; Sc strong; M_1_ between r-m and dm-cu longer than dm-cu. Calypter yellowish, with dense brownish microtrichae, margin with thin and long setulae. Knob of halter brownish, stalk yellow.

Abdomen blackish brown with grey microtomentum. Setae and setulae on abdomen black; TII-TV with setae, marginal setae longer than others; sternites with sparse black setulae at posterior 3/4. Posteromedial triangular projection of TI into TII present; SII generally luniform, the apex blunt and round, SIII irregularly oblong, SIV generally quadrate, SV depressed panduriform, apical margin arched. Male genitalia (Figs 3–4): epandrium with eight strong black setae; upper blade of bifurcated tip of surstylus swollen and apical margin rounded, lower blade thin and longer than upper one; cercus arched with short sparse setae.

*Female*. Unknown.

##### Material examined.

*Holotype*, ♂, China, Guangxi, Fangchenggang, Shangsi County (21°53'47.61"N, 107°49'20.32"E; 450 m), 18. V. 2012, Guo-Quan Wang (CAU). *Paratypes*, 2 ♂♂, same data as holotype.

##### Distribution.

China (Guangxi).

##### Etymology.

The specific name guangxiensis is derived from the type locality.

##### Remarks.

This species is similar to *P. nudipalpis* Malloch, but can be separated by the gena being approximately one-fourteenth of the eye height and the knob of the halter brownish; in *P. nudipalpis*, the gena is approximately one-eighth of the eye height and the knob of the halter is yellowish white (Malloch 1914b).

#### Phyllomyza luteigenis sp. n.

Taxon classificationAnimaliaDipteraMilichiidae

http://zoobank.org/A5CA19E7-9E6F-4EB3-8CB2-E8E0D57B559F

Figs 5–8

##### Diagnosis.

Gena approximately one-quarter of eye height. Surstylus with upper blade of bifurcated tip extremely swollen, lower blade short and thinner than upper blade.

##### Description.

*Male*. Body length 1.7–1.9 mm; wing length 1.6–1.8 mm.

**Figures 5–8.**
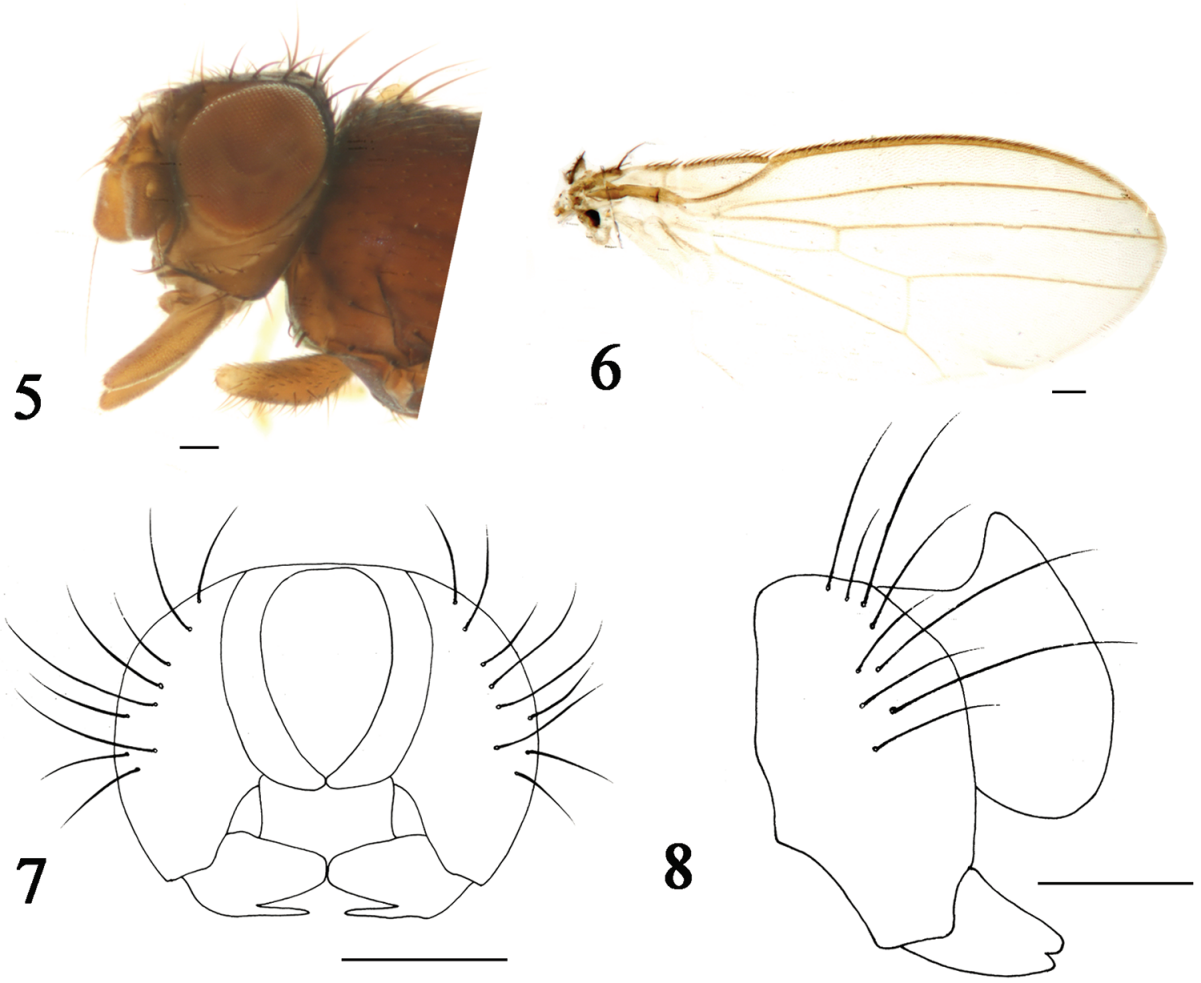
*Phyllomyza luteigenis* sp. n. (male). **5** Head, lateral view **6** wing **7** epandrium, cerci, and surstyli, posterior view **8** epandrium, cerci, and surstyli, lateral view. Scale bars: 0.1 mm.

Head (Fig. 5) darkish yellow with greyish microtomentum; orbital plate satiny yellow, with microtomentum; ocellar triangle brownish without microtomentum; lunule very depressed luniform, brownish with black margin. Posterior eye margin ventrally diverging from head margin; eye 1.1 times as high as long, gena approximately one-fourth of eye height. Setae and setulae on head black; ocellar triangle with two ocellar setae and three short setae; frons with three orbital and two frontal setae, orbital setae lateroclinate and frontal setae medioclinate; four interfrontal setae; postocellar setae cruciate. Lunule with two short setae. Vibrissal angle blunt, the tip almost a 90°angle; vibrissa strong, located at the level of lower margin of eye. Antenna blackish yellow; pedicel with black setulae at middle and margin, setulae at margin longer than others, longest one approximately 3 times longer than others; first flagellomere with pubescence, irregularly quadrate and margin blunt; arista 2.5 times as long as first flagellomere. Proboscis short and folded, brownish, with short sparse black setulae. Palpus slightly flat, 0.4 mm, apex blunt in lateral view, 3 times longer than wide; darkish yellow with short dense brownish pubescence, margin without short sparse setulae.

Thorax yellow with grey microtomentum, except scutum shiny darkish yellow with sparse brownish microtomentum; scutellum brownish yellow with gray microtomentum. Setae and setulae on thorax black; one h, two dc, one prsc, two npl, one prs, one pos, one sa, one ia, two pa, one kepsts (a row of setulae at forward position); scutellum 1.3 times wider than long, with pair of asc and bsc, asc two times longer than bsc. Legs slender, coxae and femora brownish, tibiae brownish except fore tibia darkish yellow, tarsi yellowish. Setae and setulae on legs black. Mid tibia with a single black preapical dorsal seta. Wing (Fig. 6) hyaline, unspotted; veins brown; Sc strong; M_1_ between r-m and dm-cu a little longer than dm-cu. Calypter yellowish, with yellowish microtrichae, margin with brownish setulae. Knob of halter white, stalk yellowish.

Abdomen brownish with gray microtomentum. Setae and setulae on abdomen black; TII-TV with setae at posterior 3/4, marginal setae longer than others; sternites with sparse setulae. Posteromedial triangular projection of TI into TII present; SII irregularly luniform, apical margin blunt, SIII irregularly oblong, SIV very broadly panduriform, basal margin a little wider than apical margin, SV very shallowly falciform. Male genitalia (Figs 7–8): epandrium with nine strong black setae; surstylus with upper blade of bifurcated tip extremely swollen, lower one short and slightly thin; cercus arched with short sparse setae.

*Female*. Unknown.

##### Material examined.

*Holotype*, ♂, China, Gansu, Tianshui City, Maiji Mountain (34°23'30.31"N, 106°06'35.61"E; 150 m), 15. VII. 2012, Li-Hua Wang (CAU). *Paratypes*, 3 ♂♂, China, Gansu, Tianshui City, Maiji Mountain (34°23'30.31"N, 106°06'35.61"E; 150 m), 15. VII. 2012, Ze-Hui Kang (CAU).

##### Distribution.

China (Gansu).

##### Etymology.

The specific name refers to the yellow gena.

##### Remarks.

This new species is distinctly different from other species of the genus: the gena is approximately one-fourth of the eye height, eye 1.1 times as high as long, and SV is generally very shallowly falciform.

#### Phyllomyza quadratpalpus sp. n.

Taxon classificationAnimaliaDipteraMilichiidae

http://zoobank.org/75DB8CAA-1489-4E94-875A-6B8349603DA7

Figs 9–12

##### Diagnosis.

Gena narrowed, approximately one-twelfth of eye height. Surstylus with upper blade of bifurcated tip extremely swollen, lower one slightly swollen and shorter than upper one.

##### Description.

*Male*. Body length 1.6–1.8 mm; wing length 1.6–1.8 mm.

**Figures 9–12.**
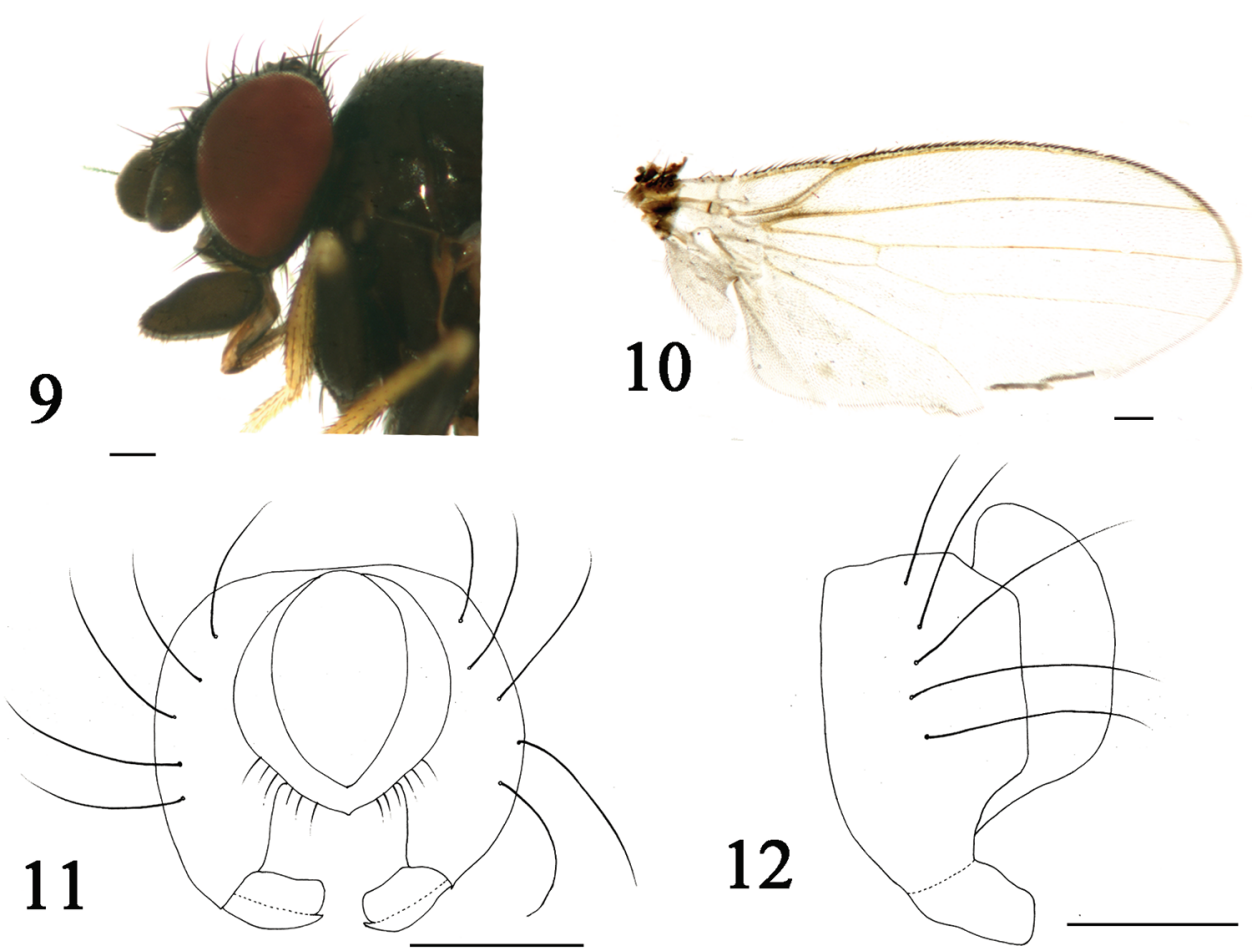
*Phyllomyza quadratpalpus* sp. n. (male). **9** Head, lateral view **10** wing **11** epandrium, cerci, and surstyli, posterior view **12** epandrium, cerci, and surstyli, lateral view. Scale bars: 0.1 mm.

Head (Fig. 9) black with grayish microtomentum; orbital plate satiny black, with microtomentum, ocellar triangle brownish without microtomentum; lunule transverse luniform, darkish brown with black margin. Posterior eye margin ventrally diverging from head margin; eye 1.4 times as high as long, gena approximately one-twelfth of eye height. Setae and setulae on head black; ocellar triangle with two ocellar setae and three short setae; frons with three orbital and two frontal setae on brownish orbital stripe, orbital setae lateroclinate and frontal setae medioclinate, four interfrontal setae; postocellar setae converging. Lunule with two short setae. Vibrissal angle flat, the tip almost a 90°angle; vibrissa strong, located at level of lower margin of eye. Antenna brownish brown; pedicel with black setulae at middle and margin, setulae at margin longer than others, longest one 4.5 times longer than others; first flagellomere with pubescence, shallowly oblong and margin blunt; arista 2.5 times as long as first flagellomere, black, distinctly pubescent. Proboscis thick and geniculate, 0.4 mm, brownish, with short sparse black setulae. Palpus flat, irregularly quadrate in lateral view, 2.5 times longer than wide; darkish brown with short dense black pubescence, margin with short sparse setulae.

Thorax darkish brown with grey microtomentum, except scutum shiny blackish brown with sparse black microtomentum; scutellum dark brown with grey microtomentum. Setae and setulae on thorax black; one h, two dc, one prsc, two npl, one prs, one pos, one sa, one ia, one pa, one kepsts (a row of setulae at forward position); scutellum 1.3 times wider than long, with pair of asc and bsc, asc three times longer than bsc. Legs slender, coxae and femora dark brown, tibiae brownish yellow except hind tibia darkish brown, tarsi yellowish. Setae and setulae on legs black. Mid tibia with a black preapical dorsal seta. Wing (Fig. 10) hyaline, unspotted; veins brown; Sc strong; M_1_ between r-m and dm-cu a little longer than dm-cu. Calypter yellowish, with brownish microtrichae, margin with brownish setulae. Knob of halter yellowish white, stalk yellowish.

Abdomen brown with grey microtomentum. Setae and setulae on abdomen black; TII-TV with setae at posterior 3/4, marginal setae longer than others; sternites with sparse setulae. Posteromedial triangular projection of TI into TII present; SII generally luniform, SIII oblong, apical margin blunt and round, SIV very broadly obpanduriform, SV shallowly oblong. Male genitalia (Figs 11–12): epandrium with five strong black setae; surstylus with upper blade of bifurcated tip extremely swollen, lower one slightly swollen and shorter than upper one; cercus irregularly arched with short sparse setae.

*Female*. Body length 1.8–2.0 mm; wing length 1.8–2.0 mm.

Similar to male, but palpus shorter, approximately four-fifths of males’. Female terminalia: TVIII brown, shallowly oblong, margin with setulae. Supra-anal plate broadly trullate; SVIII very shallowly luniform, subanal plate wide, brownish, very depressed trullate. Cercus with long setulae.

**Figures 13–18.** **13** *Phyllomyza angustigenis* Xi et Yang (male); head, lateral view **14** *Phyllomyza angustigenis* Xi et Yang (male); epandrium, cerci, and surstyli, posterior view **15** *Phyllomyza claviconis* Yang (male); head, lateral view **16** *Phyllomyza claviconis* Yang (male); epandrium, cerci, and surstyli, posterior view **17** *Phyllomyza cuspigera* Xi et Yang (male); head, lateral view **18** *Phyllomyza cuspigera* Xi et Yang (male); epandrium, cerci, and surstyli, posterior view. Scale bars: 0.1 mm.

**Figures 19–24.**
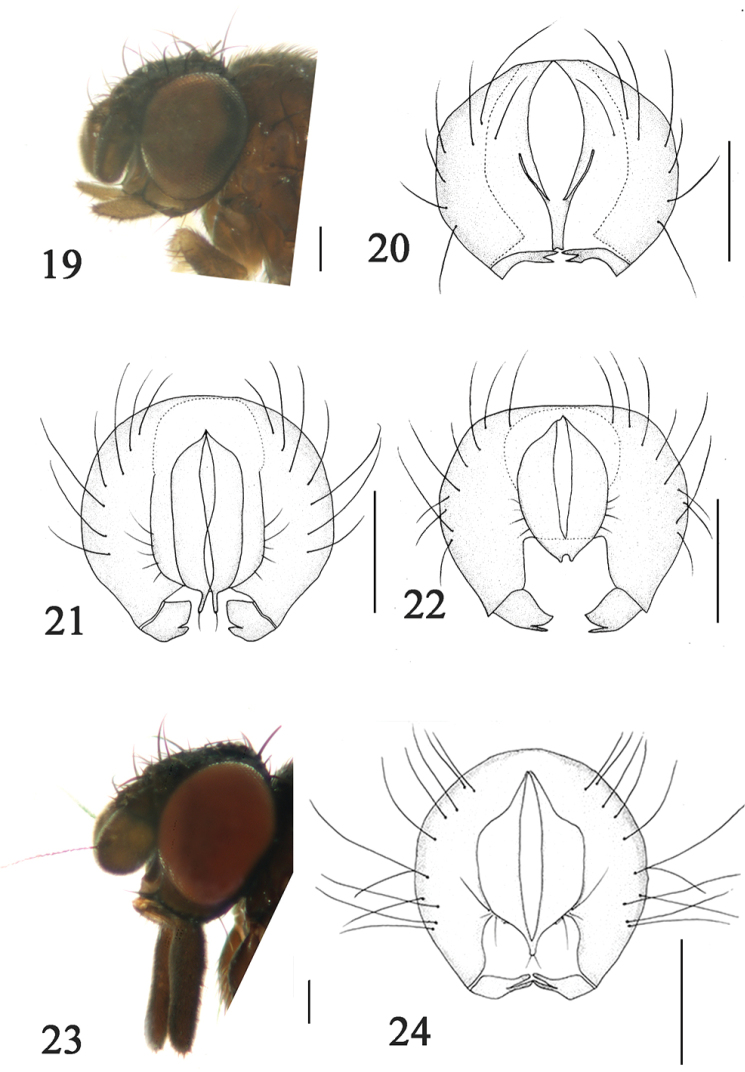
**19** *Phyllomyza dicrana* Xi et Yang (male); head, lateral view **20** *Phyllomyza dicrana* Xi et Yang (male); epandrium, cerci, and surstyli, posterior view **21** *Phyllomyza dilatata* Malloch (male); epandrium, cerci, and surstyli, posterior view **22** *Phyllomyza epitacta* Hendel (male); epandrium, cerci, and surstyli, posterior view **23** *Phyllomyza euthyipalpis* Xi et Yang (male); head, lateral view **24** *Phyllomyza euthyipalpis* Xi et Yang (male); epandrium, cerci, and surstyli, posterior view. Scale bars: 0.1 mm.

**Figures 25–30.**
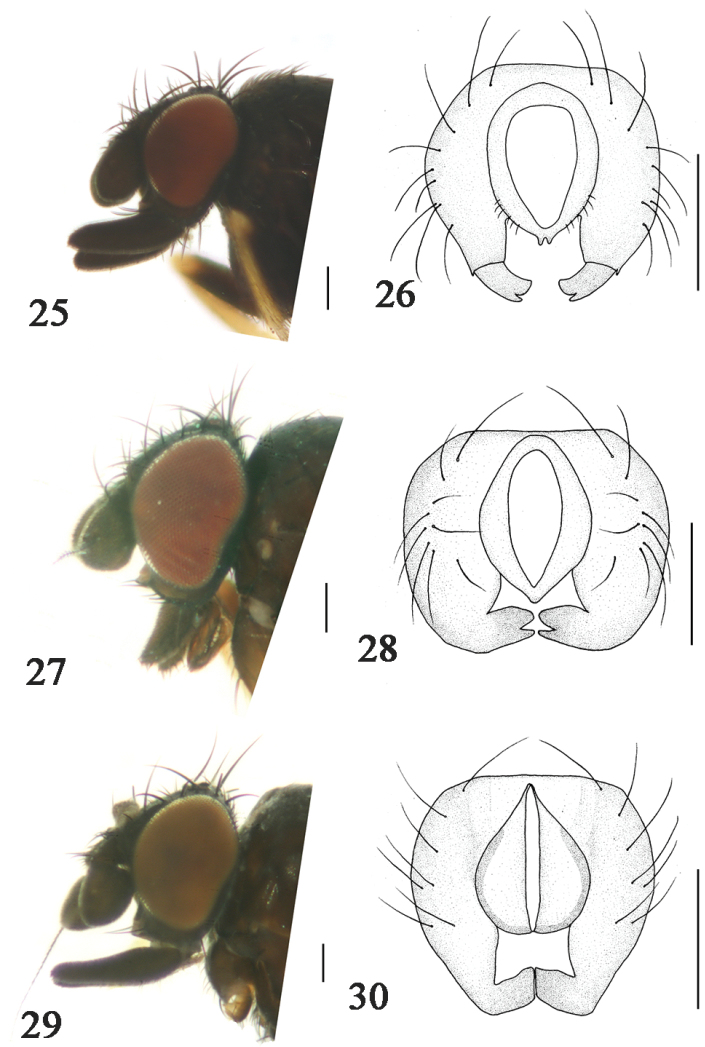
**25** *Phyllomyza nudipalpis* Malloch (male); head, lateral view **26** *Phyllomyza nudipalpis* Malloch (male); epandrium, cerci, and surstyli, posterior view **27** *Phyllomyza planipalpis* Xi et Yang (male); head, lateral view **28** *Phyllomyza planipalpis* Xi et Yang (male); epandrium, cerci, and surstyli, posterior view **29** *Phyllomyza tibetensis* Xi et Yang (male); head, lateral view **30** *Phyllomyza tibetensis* Xi et Yang (male); epandrium, cerci, and surstyli, posterior view. Scale bars: 0.1 mm.

**Figure 31.**
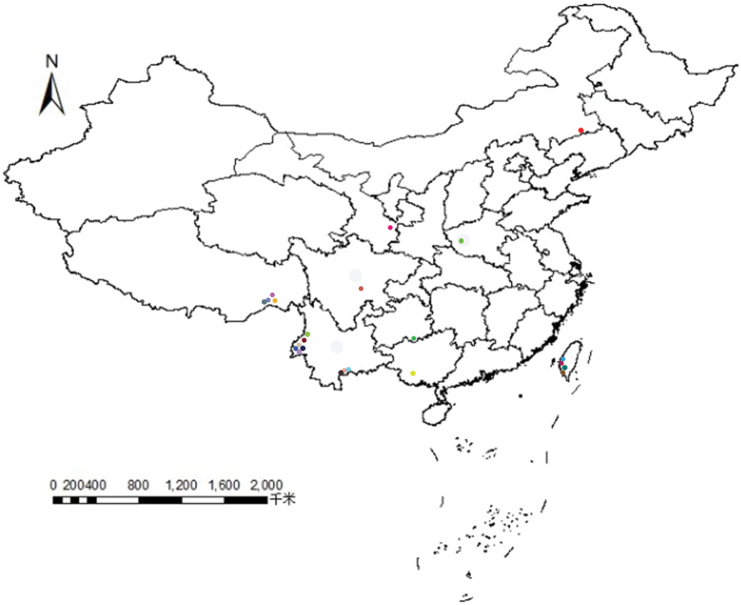
The distribution of *Phyllomyza* species in China.

##### Material examined.

*Holotype*, ♂, China, Guizhou, Libo County, Maolan National Nature Reserve (25°15'36.67"N, 108°03'21.65"E; 620m), 13. X. 2013, Ding Yang (CAU). *Paratypes*, 5 ♂♂, same data as holotype; 2 ♀♀, China, Guizhou, Libo County, Maolan National Nature Reserve (25°12'32.50"N, 108°21'20.08"E; 830m), 18. VIII. 2017, Xiaohui Hou (HAU).

##### Distribution.

China (Guizhou).

##### Etymology.

The specific name refers to the shaped of palpus.

##### Remarks.

This new species is somewhat similar to *P. planipalpis* Xi & Yang, but differs in the palpus irregularly quadrate in lateral view, surstylus with upper blade of bifurcated tip extremely swollen and apical margin smooth; in *P. planipalpis*, the palpus depressed semiluniform in lateral view, surstylus with upper blade of bifurcated tip swollen and apical margin blunt (Xi and Yang 2015a).

## Discussion

*Phyllomyza guangxiensis* sp. n. and *P. quadratpalpus* sp. n. are separately distributed in Guangxi and Guizhou Provience, this is the first reported species of *Phyllomyza* species in the two places. The palpus of *P. guangxiensis* sp. n. is wide and the basally curved, the shaped of the palpus is different with other species and cercus almost as wide as epandrium in lateral view. *P. quadratpalpus* sp. n. has a distinctive palpus, as the shaped of palpus is very shallowly rhombiod in lateral view. In Milichiidae, as far as we know, there is no similar species to *P. luteigenis* sp. n., when you consider the body colour, and palpus and eye shapes. There are 49 species of *Phyllomyza* distributed in the world, until now, 23 species are known to occur in China. Only three species are distributed in the Palaearctic Region, *P. claviconis*, *P. latustigenis*, and *P. luteigenis* sp. n., which have one similar character, which is the wide gena: *P. luteigenis* sp. n. is wider than others, and the species of Oriental Region do not have this obvious character. Twenty species are distributed in the Oriental Region, of which nine are distributed in Yunnan Province (*P. angustigenis*, *P. aureolusa*, *P. basilatusa*, *P. clavellata*, *P. cuspigera*, *P. dicrana*, *P. euthyipalpis*, *P. fuscusa*, *P. leioipalpus*) and four species in Taiwan Province (*P. dilatata*, *P. epitacta*, *P. luteipalpis*, *P. nudipalpis*) (Hendel 1914; Malloch 1914b; Xi and Yang 2013, 2015a; Xi et al. 2016) (Fig. 31). The Chinese fauna of Milichiidae is extraordinarily rich, with the continued discovery and description of further species providing great potential.

## Supplementary Material

XML Treatment for Phyllomyza guangxiensis

XML Treatment for Phyllomyza luteigenis

XML Treatment for Phyllomyza quadratpalpus
